# Detection of Expressional Changes Induced by Intrauterine Growth Restriction in the Developing Rat Mammary Gland via Exploratory Pathways Analysis

**DOI:** 10.1371/journal.pone.0100504

**Published:** 2014-06-23

**Authors:** Lea Beinder, Nina Faehrmann, Rainer Wachtveitl, Ilona Winterfeld, Andrea Hartner, Carlos Menendez-Castro, Manfred Rauh, Matthias Ruebner, Hanna Huebner, Stephanie C. Noegel, Helmuth G. Doerr, Wolfgang Rascher, Fabian B. Fahlbusch

**Affiliations:** 1 Department of Pediatrics and Adolescent Medicine, University of Erlangen-Nürnberg, Erlangen, Germany; 2 Department of Gynecology and Obstetrics, University of Erlangen-Nürnberg, Erlangen, Germany; INRA, France

## Abstract

**Background:**

Intrauterine growth restriction (IUGR) is thought to lead to fetal programming that in turn contributes to developmental changes of many organs postnatally. There is evidence that IUGR is a risk factor for the development of metabolic and cardiovascular disease later in life. A higher incidence of breast cancer was also observed after IUGR. This could be due to changes in mammary gland developmental pathways. We sought to characterise IUGR-induced alterations of the complex pathways of mammary development at the level of the transcriptome in a rat model of IUGR, using pathways analysis bioinformatics.

**Methodology/Principal Findings:**

We analysed the mammary glands of Wistar rats with IUGR induced by maternal low protein (LP) diet at the beginning (d21) and the end (d28) of pubertal ductal morphogenesis. Mammary glands of the LP group were smaller in size at d28, however did not show morphologic changes. We identified multiple differentially expressed genes in the mammary gland using Agilent SurePrint arrays at d21 and d28. In silico analysis was carried out using Ingenuity Pathways Analysis. In mammary gland tissue of LP rats at d21 of life a prominent upregulation of WT1 and CDKN1A (p21) expression was observed. Differentially regulated genes were associated with the extracellular regulated kinase (ERK)-1/-2 pathway. Western Blot analysis showed reduced levels of phosphorylated ERK-1/-2 in the mammary glands of the LP group at d21. To identify possible changes in circulating steroid levels, serum LC-Tandem mass-spectrometry was performed. LP rats showed higher serum progesterone levels and an increased corticosterone/dehydrocorticosterone-ratio at d28.

**Conclusions/Significance:**

Our data obtained from gene array analysis support the hypothesis that IUGR influences pubertal development of the rat mammary gland. We identified prominent differential regulation of genes and pathways for factors regulating cell cycle and growth. Moreover, we detected new pathways which appear to be programmed by IUGR.

## Introduction

Epidemiological studies showed that intrauterine growth restriction (IUGR) is a risk factor for the development of adult disease: An increased incidence of cardiovascular disease [1], insulin resistance, type-2 diabetes [2] and obesity [3] was detected in individuals after IUGR.

In humans, epidemiological data indicate a relation between birth weight and breast cancer risk [4]–[6]. Interestingly, studies of individuals who were in utero during the Dutch Hunger Winter suggest a 5-fold increase in breast cancer incidence following malnutrition during early gestation [7]. Maternal low protein diet (LP) is a frequently used animal model for the induction of intrauterine growth restriction. Using this model we [8] and others [9], [10] were able to identify a series of changes in several organs such as liver, pancreas, kidney and adipose tissue [reviewed in [11]]. Fernandez-Twinn et al. [12] showed that female offspring which were kept on a low protein diet until puberty, have a significantly increased incidence of early-onset mammary adenocarcinomas following nitrosomethylurea (NMU) treatment. This finding supports the notion that fetal and possibly early postnatal environment plays an important role for the developmental initiation of mechanisms underlying the programming of adult disease [13], [14]. Interestingly, the birth weight of individuals with the highest associated risk of breast cancer in the Dutch hunger study was not significantly altered [7], which might underscore the role of early-nutrition during a critical window of organ plasticity (rather than birth weight itself) as a possible mechanism of breast cancer carcinogenesis following intrauterine malnutrition. Furthermore, postnatal high caloric food intake could act as an independent, yet additive risk factor: Sloboda et al. [15], who studied the influence of a mismatched pre- and postnatal nutritional environment on pubertal development in the rat, revealed that the intrauterine environment contributes the most to changes in postnatal development. Nevertheless, postnatal overnutrition showed a moderate accelerating effect on puberty in IUGR offspring, although to a lesser degree than the intrauterine nutritional history. It is known, that breast stem cells form the mammary gland anlage in utero and remain undifferentiated until puberty, where nutritional effects are becoming evident for the first time [16]. Further investigations by Fernandez-Twinn et al. [17] however revealed that the in utero (LP diet) and postnatal environments (high calorie intake after puberty) act independently and additively to contribute to the increased breast cancer risk following intrauterine LP diet.

We sought to uncover possible molecular pathways that could give us insight into the intrauterine changes associated with this phenomenon. As ductal mammary morphogenesis is a period of fast tissue development, intrauterine alterations of molecular pathways may lay the ground for future disease of the mammary gland. We hypothesized that intrauterine malnutrition leads to reprogramming of molecular pathways in the mammary gland that can be traced during pubertal organ development. In the rat, the development of the mammary gland is known to closely resemble human pubertal mammary development due to its ducto-alveolar ultra-structure [18]–[20]. Therefore, our study was performed in a rat model of maternal LP diet. Taking into account the complex nature of mammary development [21], we chose gene expression microarray technique for the analysis of IUGR-induced changes to identify potential tissue responses in several signalling pathways at once. We acquired data from mammary tissue at the beginning (day 21) and the end (day 28) of ductal mammary morphogenesis, following maternal LP diet. Subsequently, gene expression results were linked to underlying biological processes using in silico functional pathway analysis (Ingenuity Pathways Analysis (IPA), Ingenuity Systems, www.ingenuity.com). The presented data show that intrauterine protein restriction leads to transcriptional changes in the developing mammary gland as early as day 21.

## Methods

### Animals and Surgical Procedures

All animal procedures were performed in accordance with the NIH Guide for the Care and Use of Laboratory Animals. After evaluation by the local government's review board for animal research ethics, experiments were approved by the local government authority (Regierung von Mittelfranken, approval number AZ # 54-2532.1-31/09). Surgery was performed under isoflurane anesthesia. All efforts were made to minimize suffering. Following veterinarian judgment, buprenorphine hydrochloride was injected when pain or discomfort was imminent. Our animal model was previously described in detail [8], [22]. Virgin female Wistar rats were obtained from Charles River Laboratories International, Inc. (Sulzfeld, Germany) and housed at the University's animal facility. They were maintained at 22°C on a 12h light-dark circle. Before mating, the animals had free access to standard chow and tap water. Weighing 240–260 g, they were mated and the beginning of gestation was determined via assessment of vaginal plug expellation. The pregnant dams were then randomized into two dietary groups, normal and low protein (casein) (NP and LP respectively). Throughout pregnancy animals were fed a semi-purified diet, as previously described [22]: NP rats (n = 8) received 25 g/d of Altromin C1000 containing 17.0% protein, while LP animals (n = 8) were fed 25 g/d of Altromin C1003 containing 8.8% protein. Diets were isocaloric. Contents of minerals, especially sodium (0.02%), vitamins, fat (NP, 5%; LP, 6.1%), disaccharides (11%), starch (NP, 47%; LP, 48%), methionine (NP, 10 mg/kg; LP, 8.7 g/kg), folate (10 mg/kg), and the ratio of other amino acids were similar. Rats delivered spontaneously and the litters were immediately reduced to six female pups per dam to guarantee equal lactation conditions. Postnatally, the two groups kept their label in relation to the intrauterine diets of their mothers (NP, LP). During lactation (day 1 – day 21), rat mothers were fed standard chow (17% protein content) and tap water ad libitum. The offspring was nursed by their mothers until weaned at day 21 to standard chow. At day 21 and day 28 three dams per litter were sacrificed under isofluran (Abbott 100% (V/V)) anesthesia. The lumbar mammary glands (4th and 5th pair) were collected as described by Ruan et al [23]. After removal, mammary glands were first cleared of visible lymph nodes before snap-freezing in liquid nitrogen. Samples were stored at −80°C until further processing. Gender verification was carried out via SRY gene PCR as previously described [24] and verified via ovarectomy at the day of sacrifice.

### Gene array Analysis

RNA extraction and gene array preparation were commercially outsourced to MACS molecular service at Miltenyi Biotec GmbH (Bergisch Gladbach, Germany). Mammary gland samples (n = 3 per timepoint) were shipped on dry ice. Total RNA was extracted using a standard guanidine–thiocyanate acid phenol protocol (TRIzol; Sigma-Aldrich, Taufkirchen, Germany). RNA quality and integrity were determined using the Agilent RNA 6000 Nano Kit on the Agilent 2100 Bioanalyzer (Agilent Technologies, Böblingen, Germany). RNA was quantified by measuring A260 nm on the ND-1000 Spectrophotometer (NanoDrop Technologies, Peqlab Biotechnologie GmbH, Erlangen, Germany).

Sample labeling was performed as detailed in the “One-Color Microarray-Based Gene Expression Analysis protocol (version 6.5, part number G4140-90040). Briefly, 100 ng of each total RNA samples was used for the amplification and labeling step using the Agilent Low Input Quick Amp Labeling Kit (Agilent Technologies). Yields of cRNA and the dye-incorporation rate were measured with the ND-1000 Spectrophotometer (NanoDrop Technologies). The hybridization procedure was performed according to the “One-Color Microarray-Based Gene Expression Analysis protocol (version 6.5, part number G4140-90040) using the Agilent Gene Expression Hybridization Kit (Agilent Technologies). Briefly, 0.6 µg Cy3-labeled fragmented cRNA in hybridization buffer was hybridized overnight (17 h, 65 °C) to Agilent Whole Rat Genome Oligo Microarrays 8×60 K using Agilent's recommended hybridization chamber and oven. Following hybridization, the microarrays were washed once with the Agilent Gene Expression Wash Buffer 1 for 1 min at room temperature followed by a second wash with preheated Agilent Gene Expression Wash Buffer 2 (37 °C) for 1 min. The last washing step was performed with acetonitrile.

Fluorescence signals of the hybridized Agilent Microarrays were detected using Agilent's Microarray Scanner System (Agilent Technologies). The Agilent Feature Extraction Software (FES) 10.7.3.1 was used to read out and process the microarray image files. For determination of differential gene expression FES derived output data files were further analyzed using the Rosetta Resolver gene expression data analysis system [25] (Rosetta Inpharmatics LLC., Cambridge, MA, USA). The microarray data have been deposited in NCBI's Gene Expression Omnibus [26] and are accessible through GEO Series accession number GSE34136 [27].

### IPA Functional Pathway Analysis

For further pathway analysis of biological and molecular networks underlying mammary gland remodelling after IUGR we used the web-based Ingenuity Pathways Analysis tool (IPA, Ingenuity Systems, Redwood City, CA, USA, www.ingenuity.com). The possibilities and limitations of this tool were recently described in detail by Wognum et al [28] and others [29]–[31]. Using IPA we were able to compare our Agilent microarray data with the current Ingenuity Pathways Knowledge Base (IPKB) (as of 05/13/2013). Based on our list of significantly altered genes at day 21 and day 28 (NP vs. LP), IPA computed sets for the comparative analysis of generic networks (i.e. early onset mammary adenocarcinoma, mammary gland development, steroid dependence, insulin resistance and reactive oxygen species). No fold-change cut-off was applied to the Rosetta normalized output data (p<0.05).

Human, mouse and rat orthologs of each gene were included. Comparative network analysis was carried out using the web-based software BioVenn by Hulsen et al. [32]. BioVenn uses area-proportional Venn diagrams to visualize the overlap between different lists of genes exported from IPA. For specific analysis of networks only rat orthologs were considered eligible. Stringent IPA software filtering was applied (i.e. both molecules and relationships fitted the criteria “rat”), with cut-off settings of 2.0-fold (up and down) and p<0.05. For both types of analysis only database content at the level “experimentally observed knowledge” was rendered eligible.

### Western blotting

Tissue of LP and NP rat mammary glands (n = 7 for each, litters from different mothers) was homogenized in 500 µl buffer per sample, consisting of 50 mM Hepes pH 7.4, 1% Triton X-100, 150 mM NaCl, 1 mM EDTA, 10% Glycerol, 20 µl/ml proteinase inhibitor (1 tablet Complete proteinase inhibitor dissolved in 2 ml H2O, Santa Cruz Biotechnology Inc., Heidelberg, Germany) and 2 mM sodium vanadate. Protein concentration was determined by a protein detection kit (Pierce, Rockford, IL, USA). Protein samples containing 25 µg of total protein were denatured by boiling at 95°C for 10 min and separated on a 10% denaturing SDS-PAGE gel. Electro-blotting was performed using the semidry method onto PVDF membranes (Bio-Rad Laboratories, Hercules, USA), which were then blocked with Rotiblock (Roth, Karlsruhe, Germany) for 100 min and incubated overnight at 4°C with a polyclonal rabbit anti-rat antibody to p42/44 (Cell Signaling, Danvers, MA, USA) at a concentration of 1∶1000, respectively with a monoclonal IgG rabbit anti-rat antibody to phospho p42/44 (Cell Signaling) in the concentration 1∶1000, followed by incubation for 70 min at room temperature with a secondary donkey anti-rabbit antibody (GE Healthcare, Solingen, Germany) in the concentration 1∶10000. Secondary antibodies were visualized using the fluorescent ECL+ system according to the manufacturer's instructions (GE Healthcare) and quantified with a luminescent imager (LAS-1000, Fujifilm, Berlin, Germany) and Aida image analysis software (Raytest, Berlin, Germany). Loading of the blot was quantified by staining with Amido Black [33].

### Isolation of mRNA and Real-time PCR

The frozen tissue was homogenized and total RNA was extracted using the RNeasy Midi Kit (Qiagen) with subsequent purification by additional DNase treatment using the RNeasy Mini Kit (Qiagen) both according to the manufacturer's instructions. RNA concentration was determined by NanoDrop spectrophotometry (Peqlab) and adjusted to 100 ng/ µl. First-strand cDNA was synthesized with the QuantiTect Reverse Transcription Kit (Qiagen) using random hexamers as primers. Reactions without Quantiscript Reverse Transcriptase were used as negative controls for genomic DNA contamination. Quantitative real-time PCR was performed with SYBR Green or TaqMan reagents (both Applied Biosystems, Darmstadt, Germany) using the Sequence Detector StepOnePlus (Applied Biosystems). Primers and probes are listed in Table 1. Samples were run in triplicates and mRNA levels were normalized to the housekeeping genes 18S rRNA and cytokeratin (CK)-14.

**Table 1:** Primers and probes.

Name		Sequence
r18S	forward	5′-TTGATTAAGTCCTGCCCTTTGT-3′
	reverse	5′-CGATCCGAGGGCCTCACTA-3′
CDKN1a (p21)	forward	5′-ACCAGCCACAGGCACCAT-3′
	reverse	5′-CGGCATACTTTGCTCCTGTGT-3′
WT-1	forward	5′-AGGACTGCGAGAGAAGGTTTTCT-3′
	reverse	5′-TGGAATGGTTTCACACCTGTGT-3′
CK-14	taq	5′-AGACAGAGGAGCTGAACCGCGAGGT-3′
	forward	5′-TGCCGAAGATTGGTTCTTCAC-3′
	reverse	5′-GCACCAGTTCGCTGTTGGT-3′

### Mammary gland whole mount analysis

Mammary gland whole mount analysis was carried out as described by Kleinberg et al. [23]. All chemicals came from Merck (Darmstadt, Germany), unless otherwise stated. After harvesting, tissues were fixed in ice-cold 4% paraformaldehyde for 2 h. Mammary glands were stored in 70% ethanol until further analysis. In short, following 3 washes with aceton for 30 min, mammary glands were rehydrated in 100% and 95% ethanol (30 min each). The glands were stained for 1 h with filtered hematoxylin, followed by thorough rinsing with tap water. Background staining was minimized by incubation (3×35 min) in a solution of 150 ml 100% ethanol, 150 ml aqua dest. and 7.5 ml HCl 1N (Sigma-Aldrich Chemie GmbH, Munich, Germany). Subsequently tissues were dehydrated in 70%, 95% and 100% ethanol (2×30 min for each step), followed by an incubation in Xylol (Roth, Karlsruhe, Germany) overnight. For long term storage mammary gland whole mounts were placed in methylsalicylate. Digital images of squash preparations were taken by a transmitted-light microscope stand mounted with a NIKON D200 camera (Nikon GmbH, Düsseldorf, Germany) at a distance of 60 cm all in one day. Picture analysis was performed using MetaVue software (MetaVue, Universal Imaging Corp., Downingtown, PA, USA) and ImageJ [34]. Pixel size was 11.125 µmx11.125 µm and pixel area was 123.766 µm^2^.

### WT1 Immunohistochemistry

After 4 h fixation in methyl-carnoy solution (60% methanol, 30% chloroform, 10% acid ethanol) at 4°C, tissues were dehydrated by increasing concentrations of methanol, followed by 100% isopropanol. After embedding in paraffin, 2 µm sections were cut with a Cool-Cut HM 335 E microtome (Microm international, Walldorf, Germany) and mounted on superfrost slides (Menzel, Braunschweig, Germany). Washing of the tissue sections between the different steps was performed in TBS/T. After deparaffinization, endogenous peroxidase activity was blocked with 3% H_2_O_2_ in methanol for 20 min followed by 30 min in 1% BSA/TBS both at room temperature. Sections were then layered with the primary WT1 antibody (dilution in TBS 1∶250; Thermo Scientific, Fremont, USA) and were incubated at 4°C overnight. After addition of the secondary antibody (dilution 1∶500; biotin-conjugated, goat anti-rabbit immunoglobulin G; Vector Laboratory, Burlingame, CA, USA), the sections were incubated with avidin-biotinylated horseradish peroxidase complex (Vector Laboratories, supplied by Biozol Diagnostica GmbH, Eching, Germany). Vectastain DAB (diaminobenzidine tetrahydro-chloride) kit (Vector Laboratories) served as chromogen. Each slide was counterstained with hematoxylin. Cells with positive immunoreactivity for WT1 stained brown, while WT1 negative cells showed a blue hematoxylin counterstain. We analysed the amount of WT1 positive epithelial cells per ductal area (luminal cells), after subtraction of the lumen. The surrounding myoepithelium was not included in the analysis. Graphical processing and analysis was performed using ImageJ software [34].

### Determination of Mammary Gland Proliferation

Detection of proliferating epithelial cells was carried out by immunohistochemistry as described elsewhere [35] via proliferating cell nuclear antigen kit (PCNA kit, DAKO, Hamburg, Germany). In short, tissues were prepared as described above, but cut into 3 µm sections. After deparaffinization, endogenous peroxidase activity was blocked with 3% H_2_O_2_ in methanol for 20 min followed by 30 min 100% fetal calf serum (FCS) both at room temperature. Washes between steps were carried out with TBS. Sections were then layered with a mouse monoclonal antibody against proliferating cell nuclear antigen (PCNA, dilution in 1%TBS/Albumin 1∶50; DAKO, Hamburg, Germany) and were incubated at 4°C overnight. After 3 washes, the secondary antibody (dilution in 1%TBS/Albumin 1∶500; biotinylated anti-mouse IgG, Linaris Biologische Produkte GmbH, Dossenheim, Germany) was added for 30 min at room temperature. After 3 washes, peroxidase-coupled avidin D was added, as suggested by the manufacturer (30 min, room temperature). After 3 washes, DAB-staining was performed for 8 min. PCNA positive cell nuclei stained brown. Slides were washed with aqua dest. for 5 min and counterstained with hematoxylin for 15 sec. After alcoholic dehydration, followed by 5 min incubation in Xylol, sections were covered in Entellan (Merck). Stained sections were evaluated in a Leitz Aristoplan microscope (Leica Instruments, Wetzlar, Germany). Epithelial cells within a terminal end bud (TEB) were analyzed using ImageJ software [34]. TEBs were defined by a minimum of 3 layers of epithelial cells as seen in longitudinal sections (see Figure S1). Surrounding myofibroblasts cap cells were not included in the analysis. The number of PCNA-positive cells/TEB area in µm^2^ (pixel size was 0.243 µmx0.243 µm, which was equal to a pixel area of 0.059 µm^2^) was determined. Mammary glands of 2 animals per litter at the respective time points were examined.

### Liquid Chromatography Tandem Mass Spectrometry (LC-Tandem MS)

Steroid profiling was performed by LC-Tandem MS, as previously described [36], [37]. A detailed description of the LC-Tandem MS method can be found in Method S1.

### Statistical analysis

Statistical differences were analyzed by the non-parametric Mann-Whitney U Test using GraphPad Prism 4.0 (GraphPad Software, San Diego, CA, USA). Data are means ± standard error of the mean (SEM) and p<0.05 was considered significant.

## Results

### Auxologic data of animals

**Figure 1:**
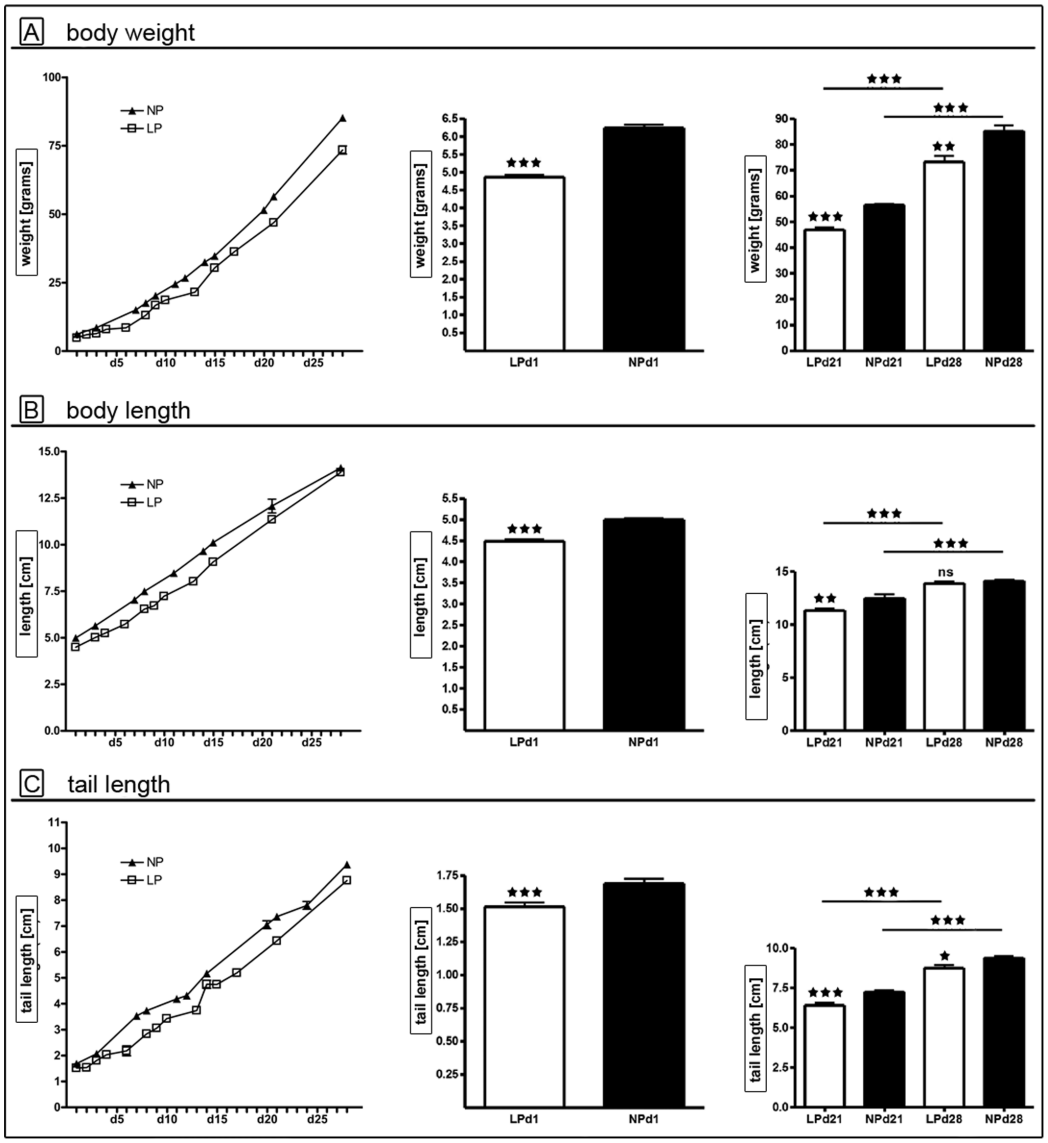
Auxologic data. Body weight (A), body length (B) and tail length (C) of the LP (white bars/squares) and NP (black bars/triangles) group during postnatal development (left), at day 1 (middle) and day 21 vs. day 28 (right). At d1 - d21 weight data was recorded from n = 40 LP and n = 47 NP animals, at d21-d28 from n = 20 LP and n = 24 NP rats. Length and tail length measurements came from n = 20 LP and n = 26 NP pups at d1 – d21, and from n = 20 LP and n = 24 NP rats at d21 – d28. LP =  low protein (white bars); NP =  normal protein (black bars); d =  day; * =  p<0.05, ** =  p<0.01; *** =  p<0.001; ns =  not significant.

We continuously monitored auxologic parameters of female LP and NP animals (Figure 1). There was no significant difference in litter size or gender distribution (data not shown). Both groups showed body growth in the course of development (p<0.0001 for day 1, day 21 and day 28). At birth the LP group was significantly growth restricted (p<0.0001, Figure 1), compared to the NP group. LP rats showed a catch-up in body length from day 21 (p = 0.001, Figure 1 B) until day 28, where body lengths of NP and LP animals did not differ any more. In contrast, body weight and tail length of LP rats remained significantly reduced at day 21 (both p<0.0001) and at day 28 (p<0.002 and p<0.03, respectively) (Figure 1 A and C).

### Mammary gland development

#### Analysis of Ductal Morphogenesis

**Figure 2:**
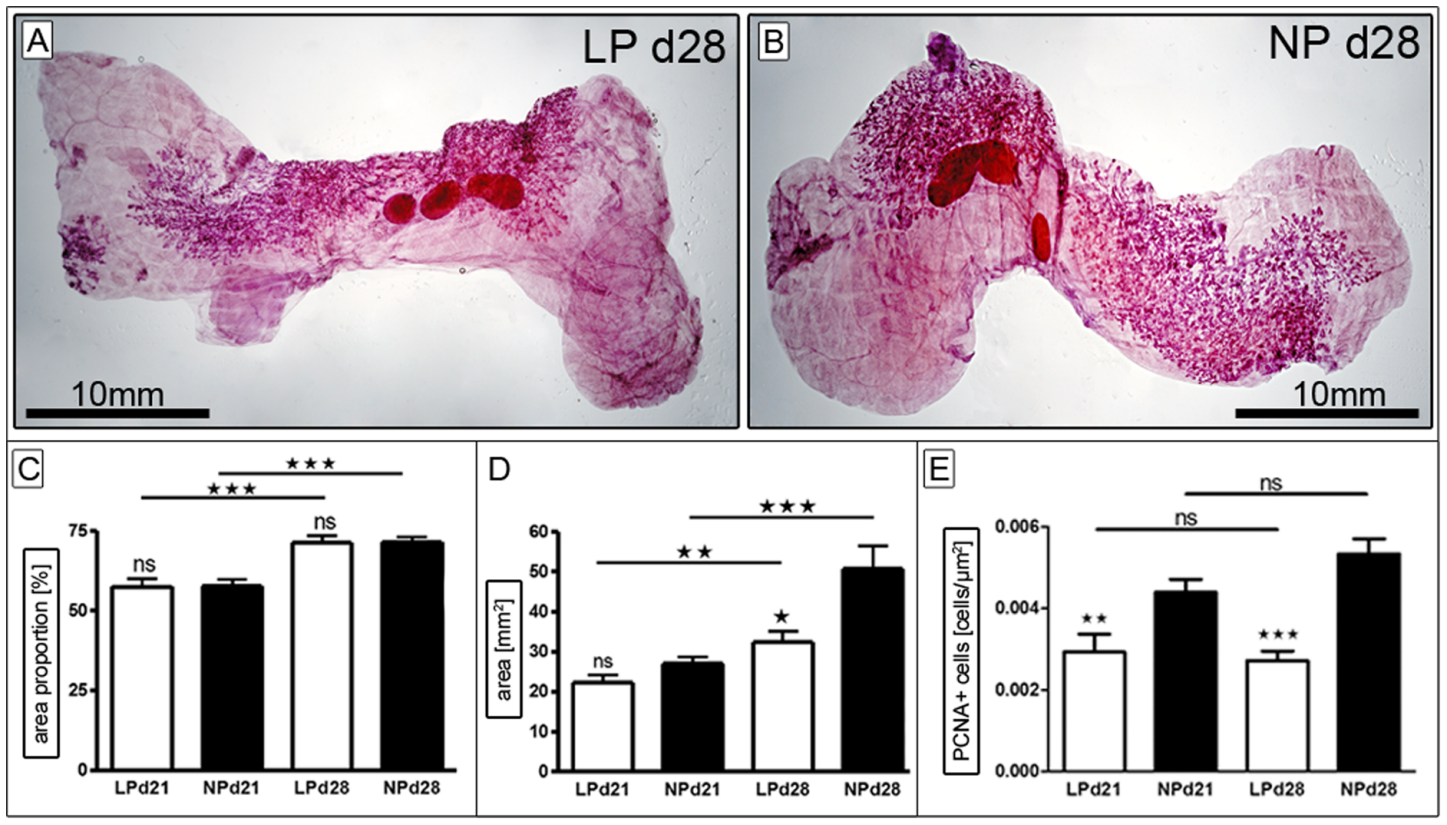
Analysis of ductal morphogenesis. A+B) Exemplary transmitted light microscope images of lumbar mammary gland whole mount preparations at day 28 of LP (A) and NP (B). Magnification is indicated by the black bar (10 mm). The percentage of lumbar mammary fat pad occupied by ducto-alveolar structures (“area proportion” in %, C), as well as the area (mm^2^) occupied by ducto-alveolar structures itself (D) were examined at day 21 (LP n = 13, NP n = 18) and day 28 (LP n = 12, NP n = 10). Furthermore the rate of proliferation was determined immunohistochemically in terminal end buds (TEB) via PCNA-stain (E, see also Figure S1), with LP d21 (n = 6), LP d28 (n = 10), and NP d21 (n = 7), NP d28 (n = 9). LP =  low protein (white bars); NP =  normal protein (black bars); d =  day; * =  p<0.05, ** =  p<0.01; *** =  p<0.001; ns =  not significant.

For the analysis of mammary gland ductal morphogenesis whole-mount preparations were performed using the apical (4^th^) and distal (5^th^) mammary gland of the conjoined lumbar mammary gland fat pad (Figure 2). We determined the area of mammary fat pad (in percent) covered by ducto-alveolar structures. At day 21 (beginning of pubertal mammary gland development/thelarche), approximately 57% of the lumbar mammary fat pad was covered by ducto-alveolar structures (Figure 2 C). This percentage significantly increased until day 28 (end of pubertal mammary development), where approximately 71% of the lumbar mammary fat pad was occupied by the glandular body. We did not detect a significant difference between the LP and the NP group at both time-points (Figure 2 C). The percentage of mammary fat pad invaded by ducto-alveolar structures did not vary between apical and distal lumbar glands, nor did we see a difference between groups when normalizing the percentage to the rat body weight (data not shown). When looking at the dimension of ducto-alveolar expansion in the mammary fat pad per se, LP animals showed a significantly smaller area of ducto-alveolar structures (p = 0.02) when compared to NP at day 28, while the glandular body was equally sized at day 21 (Figure 2 D). This significant difference persisted after normalization to body weight (data not shown). Taken together, LP animals showed a smaller area of ducto-alveolar structures at day 28 when compared to NP animals. The density of the gland (ducto-alveolar structures per area), however was equal in both groups at day 21 and day 28.

##### Proliferation rate of terminal end buds

Proliferative activity was analyzed in the proliferative core unit of the developing mammary gland: the terminal end bud (TEB) (Figure S1). The number of proliferating cells per TEB-area (µm^2^) was identified using PCNA-staining. We found significant differences at day 21 and day 28 for both groups: The proliferative activity in TEBs of LP animals was decreased by 34% at day 21 and was approximately 49% lower than the proliferation rate of TEBs in NP animals at day 28 (Figure 2 E).

### Serum Steroid-profiling

We used LC-Tandem MS for the measurement of serum steroid levels in LP and NP rats at day 21 and day 28. Normalization of steroid serum levels to auxologic parameters such as body weight and length had no influence on group differences (not displayed).

#### Corticosterone and dehydrocorticosterone

**Figure 3:**
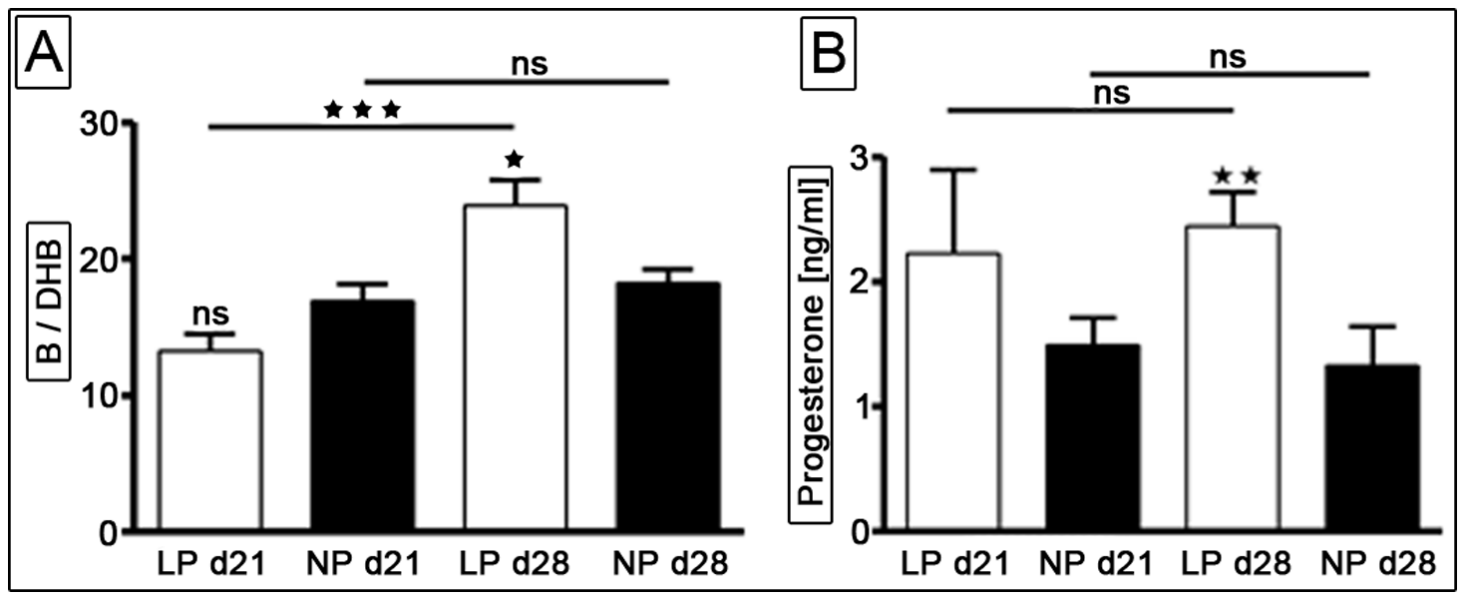
Serum steroid levels detected by Liquid Chromatography Tandem Mass Spectrometry [LC-Tandem MS]: Serum corticosterone [B] –to- dehydrocorticosterone [DHB] ratio (A) and serum progesterone levels (B) were determined at day 21 (LP n = 21, NP n = 22) and day 28 (LP n = 20, NP n = 24). Further data regarding LC-Tandem MS intensity profiles of (dehydro-) corticosterone and progesterone (e.g. multiple reaction monitoring, retention time etc.) and a detailed description of the method can be found in Method S1. LP =  low protein (white bars); NP =  normal protein (black bars); d =  day; * =  p<0.05, ** =  p<0.01; *** =  p<0.001; ns =  not significant.

Rodent active corticosterone (B) and inactive dehydrocorticosterone (DHB) correspond to active cortisol and inactive cortisone in humans. The level of B raised significantly in the LP and NP group (p<0.0001 and p = 0.02, respectively) from day 21 (LP: 169.4±24.2 ng/ml; NP: 189.0±19.1 ng/ml) to day 28 (LP: 390.0±27.0 ng/ml; NP: 300.0±32.4 ng/ml) (Figure 3 A). Analog to the B levels, DHB levels significantly increased in the LP and NP group (p = 0.002 and p<0.03, respectively) from day 21 (LP: 11.7±1.1 ng/ml; NP: 11.2±1.0) to day 28 (LP: 16.7±0.9 ng/ml; NP: 15.8±1.5 ng/ml) (Figure 3 A). There was no significant difference between B and DHB levels in both groups for day 21 and day 28. Interestingly, when analyzing the relation of active B to inactive DHB (B/DHB) LP animals showed a significant increase in active B at day 28 in comparison to the NP group. Moreover, we observed a significant increase of B/DHB quotient in LP animals from day 21 to day 28 (p<0.0001, Figure 3 A). We did not see such an increase in the NP group.

##### Progesterone

We found that both LP and NP rats maintained constant progesterone serum levels at day 21 and day 28. Interestingly, serum progesterone levels were significantly increased in the LP group at day 28 in comparison to the NP animals (p = 0.0023) (Figure 3 B).

#### Deoxycorticosterone

Deoxycorticosterone is an intermediate product in the metabolism from progesterone to corticosterone. Analog to the progesterone serum levels deoxycorticosterone levels were significantly higher in the LP group (p = 0.01) at day 28 (data not shown). In addition, deoxycorticosterone serum levels increased significantly in the LP group from day 21 to day 28 (p = 0.0389), while NP serum levels at day 28 were reduced by 16% when compared to day 21 (data not shown).

#### Dehydroepiandrosterone (DHEA) and Dehydroepiandrosterone-sulfate (DHEAS)

Serum DHEA- and DHEAS-levels were close to the detection limit at both time-points. While no inter- or intra- group differences were detected for DHEAS at day 21 and day 28, DHEA serum levels significantly decreased in the LP and NP groups at day 28 (p = 0.0025 and p = 0.0017, respectively). This decrease was more significant in the NP group, resulting in significantly higher DHEA serum levels in the LP animals at day 28 (p = 0.03) (data not shown). However, DHEA to DHEAS ratios showed no significant intra- or inter- group differences at day 21 and day 28 (data not shown).

### Gene array Analysis

#### Mammary Genes and Gene Networks post-IUGR

The microarray data at the beginning (day 21) and end (day 28) of pubertal mammary ductal morphogenesis were analyzed online using IPA software, comparing LP rats to their NP controls. For specific analysis at day 21, 337 genes were deemed to be differentially regulated (p<0.05, 2-fold), 58% of which were up-, 42% of which were down-regulated. At day 28 a total of 280 genes were found to be differentially regulated (p<0.05, 2-fold), 70% of which were up-, and 30% of which were down-regulated. Those genes were subjected to further in silico analysis, to identify the most relevant focus genes and their respective networks based on their IPA score. The networks at day 21 and day 28 that were most significantly evoked following intrauterine LP diet can be found in the supplement (Figure S2 and S3, respectively), including the top biological functions and genes underlying each network (Table S1). The top regulated networks based on IPA score were “Lipid Metabolism, Molecular Transport, Small Molecule Biochemistry”, “Cell-To-Cell Signalling and Interaction” and “Cancer, Cellular Assembly and Organization, Cellular Compromise” on day 21 and “Drug Metabolism, Lipid Metabolism, Small Molecule Biochemistry”, “Cardiovascular/Hematological System Development and Function” and “Organismal Injury and Abnormalities, Cardiovascular Disease ” on day 28.

#### IPA gene network analysis: Differentially regulated genes

We were surprised to find, that many of the top-regulated genes (as determined by IPA software in silico analysis) following intrauterine LP diet were linked to (patho-)physiologic processes of the mammary gland:. The top 10 differentially expressed genes (up- and down-regulated) in the mammary gland at day 21 and day 28 are listed in Figure S2 and S3, respectively. Of the ten top induced genes 40% at day 21 and 30% at day 28 are believed to play a role in mammary gland (patho-)physiology, while of the ten top down-regulated genes 50% at day 21 and 30% at day 28 are believed to have a functional association with mammary gland physiology. These genes are displayed in Table 2. WT1 as a top-regulated gene was chosen for further analysis, because we were previously able to show that it is differentially regulated during postnatal kidney development of male rats following IUGR [38].

**Table 2:** IPA gene network analysis: Top differentially regulated genes at day 21 and day 28 associated with mammary gland (patho-)physiology.

Day 21			
ID	Name	fold-change	p-value
POU3F3	POU domain, class 3, transcription factor 3	8.2	0.00002
GALR1	Galanin receptor type 1	7.2	0.00131
UGT2B7	UDP-glucuronosyltransferase 2 family, polypeptide B7	6.7	0.0046
WT1	Wilms tumor protein homolog	5.3	0.00796
Ugt2b	UDP-glycosyltransferase 2 family, polypeptide B	−4.1	0.014
HRG	Histidine-rich glycoprotein	−4.6	0.016
SULT2A1	Sulfotransferase family, cytosolic, 2A, dehydroepiandrosterone (DHEA)-preferring, member	−5.6	0.00000009
CYP3A4	Cytochrome P450 3A4	−6.0	0.0007
TNFRSF17	Tumor necrosis factor receptor superfamily, member 17	−6.3	0.000009
Day28			
ID	Name	fold-change	p-value
MYOD1	Myoblast determination protein 1	9.3	0.00001
PITX2	Homeobox protein PITX2	6.9	0.007
GZMB	Granzyme B	6.0	0.002
CCR4	Chemokine (C-C motif) receptor 4	−2.5	0.002
NR1D1	Nuclear receptor subfamily 1 group D member 1	−3.2	0.002
BMP-10	Bone morphogenetic protein-10	−8.6	0.0006

#### IPA gene network analysis: Comparative analysis of generic networks

**Figure 4:**
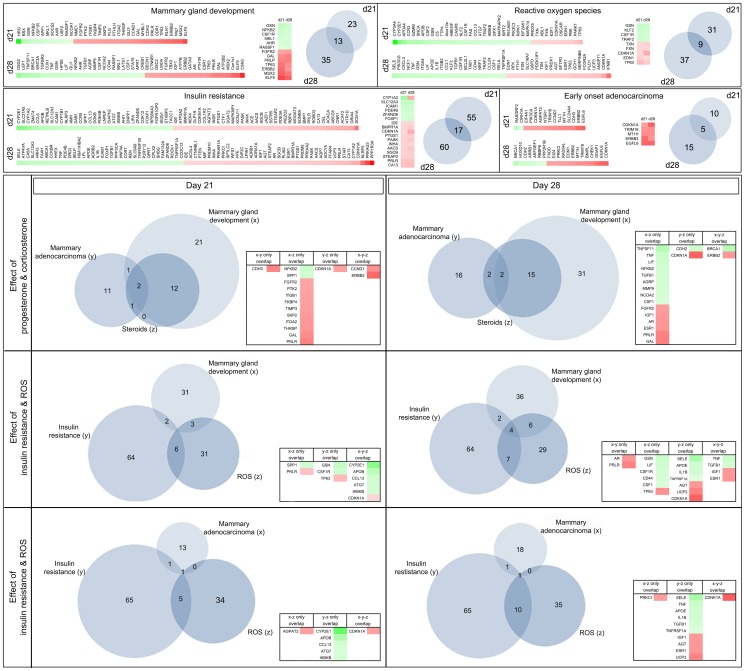
BioVenn diagram analysis of functional gene clusters: Displayed are the results of the comparative analysis of differentially regulated genes using IPA Ingenuity software and consecutive Venn diagram transformation. The upper section lists differentially regulated genes of the generic networks mammary gland development, reactive oxygen species (ROS), insulin resistance and early mammary adenocarcinoma at day 21 and day 28 as heatmaps. The lower section displays overlapping genes of these generic networks as Venn Diagrams at day 21 (on the left) and day 28 (on the right). The quantity of regulated genes of each network cluster is indicated in the respective small Venn diagrams by number. In the upper section, common genes that are differentially regulated at both day 21 and day 28 are represented by the amount of overlap of the circles and listed in the small table next to the diagram. In contrast, the tables in the lower section of this figure display overlapping genes of three selected pathways as indicated. Red =  up-regulated gene; green =  down-regulated gene; white =  fold-change value of 0.

For comparative panel analysis the software identified 2635 genes to be differentially regulated at day 21 (p<0.05) and 3431 genes at day 28 (p<0.05), 52% of which were up- and 48% were down-regulated at day 21 and 48% up- and 52% down-regulated at day 28, respectively. Those genes were subjected to further in silico analysis. Our main focus was the identification of early molecular changes in pubertal mammary development following poor fetal nutrition using a global gene array approach. Based on the findings of Fernandez-Twinn et al. [12], we were especially interested whether we could observe IUGR-induced changes in the mammary transcriptome associated with “insulin resistance” and how such changes would relate to the panels “mammary gland development”, early-onset ductal “mammary adenocarcinoma” and to the influence of corticosterone/dehydrocorticosterone, as well as progesterone (“steroids”) and reactive oxygen species (“ROS”). The resulting panels with integrated fold-change (heatmap) are displayed in Figure 4. Venn diagram based analysis showed that the panel “steroids” is a major subset of the panel “mammary gland development”, while differentially regulated genes of the panels “insulin resistance” and “ROS” barely overlapped with the developmental panel. Interestingly, CDKN1A was computed to be an important nexus gene between the panels “insulin resistance” - “ROS” - “mammary adenocarcinoma”, with an induced expression at day 21 and day 28 (2.3-fold, p<0.00001). It is worth noticing, that CDKN1A also links the panels “steroids” and “mammary adenocarcinoma”. In silico analysis further identified Erbb2 as a differentially up-regulated (2.1-fold, p<0.05 at day 21) nexus gene of “mammary adenocarcinoma” and “mammary development” at day 21 and day 28 (Figure 4).

Furthermore, analysis of the top-regulated networks revealed a central role for the extracellular signal-regulated kinase (ERK)-1/-2 as a core gene, defined by computation of multiple direct relations to differentially regulated genes (data not shown). As ERK-1/-2 activity is regulated by phosphorylation no expressional change was evident in our online array analysis. In addition, IPA software analysis computed a downregulation of transforming growth factor beta 1 (TGF-β1) as the central network regulator for cellular development in LP mammary glands at day 28, while at day 21 the TGF-β1 pathway was only slightly influenced by maternal nutrition (Figure S4).

### Verification of selected differentially expressed genes and pathways

**Figure 5:**
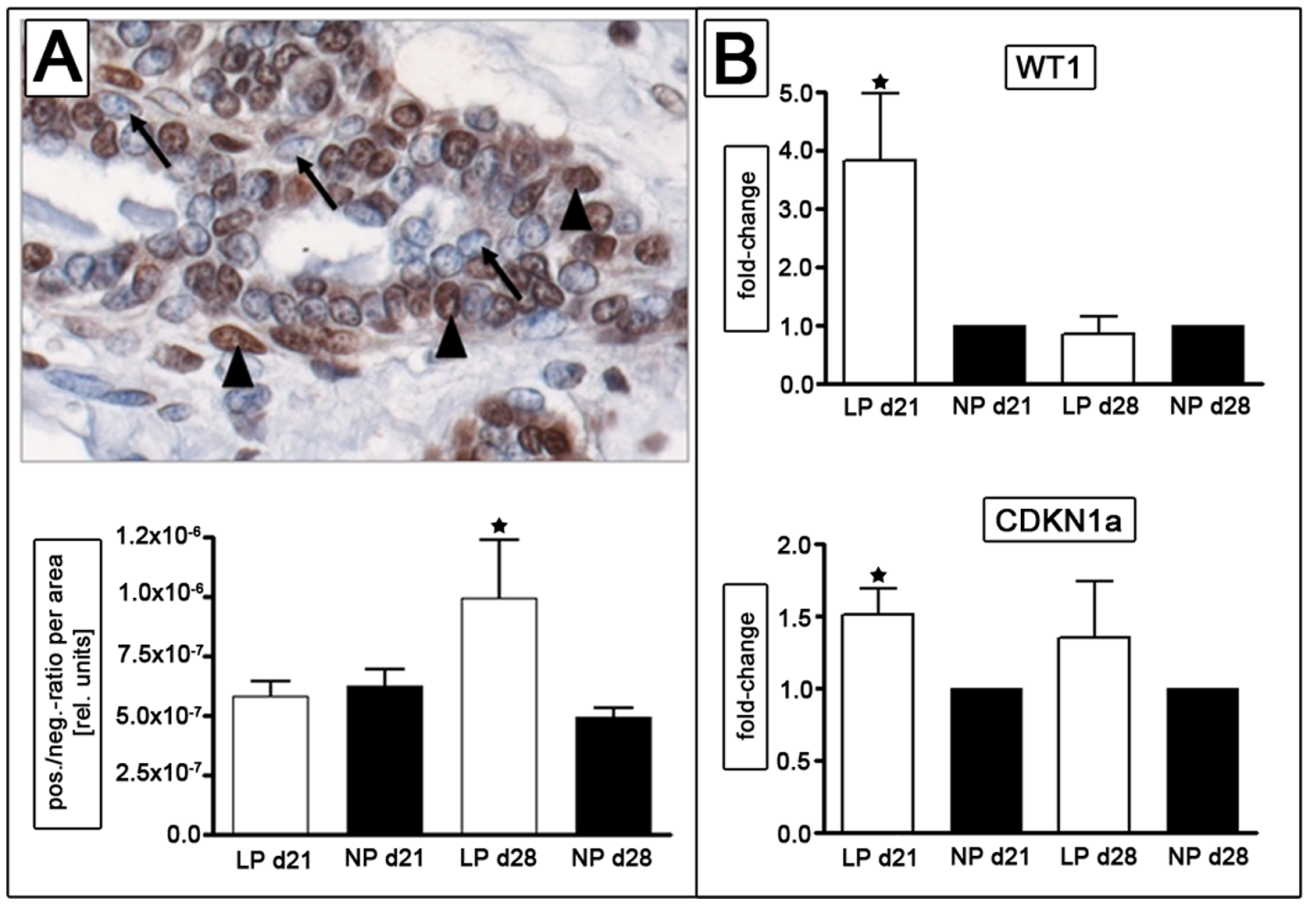
Analysis of Wilms tumor suppressor gene 1 (WT1) expression in lumbar mammary glands at day 21 and day 28. WT1 stained positive (black arrow head) in epithelial cells (A, WT1-negative cells are indicated by arrows), with a significant increase of WT1 positive cells per area at day 28 in the LP group. RT-PCR indicates a significant induction of WT1 and CDKN1a (p21) expression at day 21 (B). LP =  low protein (white bars); NP =  normal protein (black bars); d =  day; * =  p<0.05 versus NP of the same time point.

WT1 protein was highly abundant in mammary glandular structures of both groups, with higher expression in the luminal cells of the gland. We did not determine a significant difference in the ratio of WT1 positive to WT1 negative cells per area between NP and LP at day 21. At day 28 we found a significant increase (p<0.033) in WT1 positivity in the luminal cells of the LP group. The results are shown in Figure 5 A. Moreover, LP animals showed a significantly increased expression of WT1 at day 21 (3.8-fold, p<0.05) in their mammary glands by real-time PCR analysis (Figure 5 B). By real time PCR, we also verified the increase in expression of the cell cycle regulator CDKN1A in the mammary gland of LP rats (1.5-fold, p<0.04) (Figure 5 B).

**Figure 6:**
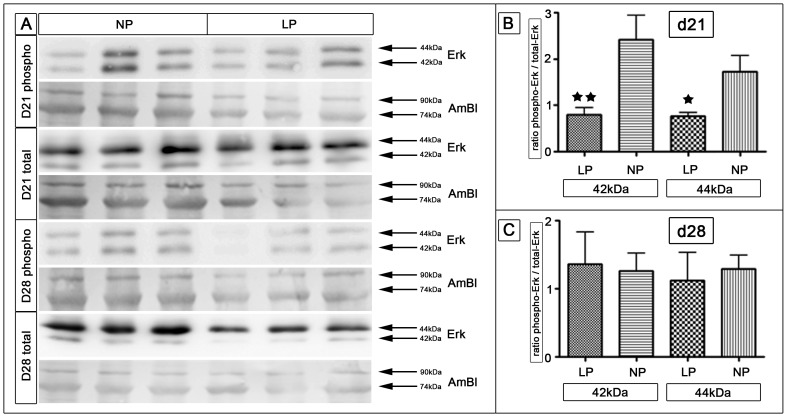
Analysis of extracellular signal-regulated kinase (ERK) -1 and -2 activities via Western blot. (A) Tissue lysates from lumbar mammary glands at day 21 and day 28 were probed with an antibody recognizing phosphorylated (activated) and total (phosphorylated and non-phosphorylated) ERK1 (also known as p44, upper band 44kDa) and ERK2 (also known as p42, lower band 42kDa). Amidoblack (Ambl) served as control. (B+C) Densitometric analysis presented as the ratio of activated ERK-1/-2 to total ERK at day 21 (B) and day 28 (C). LP =  low protein (white bars); NP =  normal protein (black bars); d =  day; * =  p<0.05, ** =  p<0.01.

In silico functional pathway analysis detected ERK-1/-2 as a central nexus protein (Figure S5). Hence we examined ERK-1/-2 activation via Western blot analysis (Figure 6 A). We found a constitutive expression of ERK-1 and ERK-2 isoforms in lysates from rat mammary glands. Activation of ERK was identified using an antibody specific for the dual-phosphorylated forms of ERK-1 and ERK-2. In comparison to NP rats, LP animals showed a significantly lower phosphorylated-to-total ratio for ERK-1 and ERK-2 (p<0.05 and p<0.01, respectively) at day 21 (Figure 6 B). Mammary glands of LP rats showed a significantly reduced ERK activation at day 21. The phosphorylation of ERK in the mammary gland of NP rats was 1.8-fold and 2.2-fold higher for ERK-1/-2, respectively (Figure 6 B). We did not detect a significant difference between both groups in ERK-1 and ERK-2 activation at day 28 (Figure 6 C).

## Discussion

The main finding of our gene array analysis in developing mammary glands of IUGR rats is an altered expression of genes involved in cell cycle and growth. This is accompanied by smaller mammary glands and reduced proliferation rates of the budding epithelium after IUGR. In silico pathway analysis ranked WT1 among the top-induced genes in the IUGR group, which was confirmed by RT-PCR and immunohistochemistry. Moreover, Venn diagram based gene-panel analysis identified CDKN1A as a significantly up-regulated nexus gene, which we confirmed by RT-PCR. In silico network analysis further showed a dysregulation of many components of the ERK-1/-2 pathways. This was accompanied by a reduced phosphorylation of ERK-1 and ERK-2 in the IUGR group at day 21. IUGR animals showed significantly increased serum hormone concentrations of progesterone and corticosterone/dehydrocorticosterone-ratio at day 28.

IUGR can be induced by a variety of animal models [39]. We chose the model of maternal low protein diet, as it has proven itself a reproducible and effective method for programming changes in organ development in male rats [38]. A model of maternal low protein diet was also used by others to examine changes in female mammary gland development [12], [17], [40]. However, postnatal treatment and observational periods vary among these studies. After its formation in utero, the rat mammary primordium remains developmentally silent [41]. In the rat prepubertal mammary gland development shows two physiologic peaks at the 28-29^th^ (ductal morphogenesis) and the 34–35^th^ day of age [16].

In the recently published data on their animal model of intrauterine malnutrition, Fernandez-Twinn et al. [12] continued the protein restriction until weaning (day 21) and monitored breast development up to day 112. They found reduced secondary ductal branching and epithelial invasion at day 21, which was followed by a rapid post-weaning compensatory mammary growth [12]. Using a similar model, Zheng et al. on the contrary switched to normal protein diet at birth and only examined molecular changes in the mammary glands at day 38, without looking at histologic changes.

To closely resemble the human pathophysiology of IUGR, we chose to change our dietary regimen to normal protein diet at birth, as described by [40]. We studied mammary gland development at its first postnatal peak (day 21 to day 28), similar to Fernandez-Twinn et al. [12], in order to identify intrauterine influences on the mammary gland anlage during the critical time of ductal morphogenesis. Our IUGR rats showed a reduced extension of ductal structures into the mammary fat pad with no further ultra-structural alterations, while in the study by Fernandez-Twinn et al. [12] ductal branching was reduced in IUGR rats. Because their animal model differs from ours in the timing of the low protein diet, we hypothesize that the developmental changes observed by Fernandez-Twinn et al. [12] might primarily result from postnatal influences rather than from fetal programming itself, as previously suggested [42]. Based on these findings, the postnatal phase seems to play a distinct role for mammary gland development following IUGR in the rat.

It has been shown, that IUGR leads to hyperglycemia, insulin resistance and type-2 diabetes [43]. Insulin resistance and type-2 diabetes are associated with an increased breast cancer risk [44]. As described by Wolf et al. [45], diabetes mellitus and breast cancer development are linked via activation of the insulin- and IGF-I pathways, as well as via the regulation of endogenous steroids. Fernandez-Twinn et al. [12] attributed the increased breast cancer incidence of their IUGR rat model mainly to mechanisms of insulin resistance. However, no changes in endogenous steroid levels were observed. In contrast, we found an increase in serum progesterone and corticosterone/dehydrocorticosterone-ratio in our female LP rats at day 28. In the rat, progesterone is enzymatically (21α-hydroxylase, 11β-hydroxylase) converted into the primary rat glucocorticoid corticosterone via the intermediate deoxycorticosterone, which was also increased in our LP rats at day 28 [46]. We did not detect differences in corticosterone serum levels in the male rat, using the same animal model [47] and method (LC-Tandem MS). Hence we hypothesize that the increase in serum corticosterone in our female IUGR rats could be secondary to the increase in progesterone.

Progesterone increases the proliferative effect of IGF-I on ductal mammary morphogenesis [48]. Physiologically, synthesis of progesterone occurs at day 38 in rat ovaries [49]. Hence, an increase of progesterone at day 28 in our LP group might reflect an acceleration of ovarian maturation. In line with our hypothesis, Sloboda et al. [42] found reduced serum progesterone levels in adult rats born IUGR and assumed a premature ovarian function with a consecutive early menopause. Similar observations were made in humans: in the Dutch-famine study Painter et al. [7] observed that IUGR in the first trimester of pregnancy is associated with an earlier reproductional phase post-partum and an earlier onset of menopause.

Earlier maturation post-IUGR has also been observed in girls, however, conflicting data exist regarding this issue (reviewed by [50]). In our study, the reproductive performance of former IUGR individuals was not determined. Alternatively, increased progesterone levels could be explained by a rapid progression of pubertal development rather than an early onset [51]. Interestingly, early menarche is associated with an increased risk of breast cancer [52]. Our gene array based Venn diagram analysis indicates that the panels “steroids” and “mammary gland development” have numerous common genes which are differentially regulated by intrauterine malnutrition. Hence, direct and indirect effects of intrauterine malnutrition on pubertal mammary gland development are conceivable, the latter could be partly related to steroidal influences. However, as progesterone particularly regulates tertiary sidebraching of epithelial ducts following ductal morphogenesis [21], its transcriptional effects might become structurally visible in older animals only.

Our Venn diagram based gene panel analysis revealed a large number of differentially regulated genes associated with insulin resistance, as suggested by Fernandez-Twinn et al. [12]. Unexpectedly, most of these genes were not shared by the panel mammary gland development. This might point to an important, yet separate role of insulin resistance in our model and could fit our observation of an ultrastructurally normal mammary gland in our IUGR rats. However, we cannot rule out an influence of genes related to insulin resistance on later phases of mammary gland development.

In IUGR insulin resistance and the development of adult sequelae are partly driven by an increased generation of reactive oxygen species (ROS) [53]. We found a certain number of differentially regulated, ROS-related genes in LP rats at day 21 and day 28, which need to be evaluated in further studies. Our in silico analysis identified the cyclin-dependent kinase inhibitor 1a (CDKN1A, also known as p21) as a gene of future interest, as it links the gene panels of “ROS”, “insulin resistance” and “mammary adenocarcinoma”. It is worth noticing, that CDKN1A additionally connects the panels “steroids” and “mammary adenocarcinoma”. It is an important regulator of mammary gland proliferation and differentiation [54]. Dependent on the cellular context CDKN1A can both promote and inhibit tumorigenesis mainly via its regulation of p53-dependent cell cycle arrest [54]. Overexpression of CDKN1A [55] leads to inflammation and impairment of insulin sensitivity, possibly mediated through increased production of ROS [56] and mitochondrial dysfunction. In a low-protein animal model similar to ours, Zheng et al. [40] demonstrated a reduced expression of CDKN1A in the mammary gland at day 38, while Fernandez-Twinn et al. [17] found an induction of CDKN1A expression in their IUGR model at 5 weeks of age. We found an induction of CDKN1A expression in our IUGR rats at day 21. The fact that CDKN1A has context specific functions might therefore point to a differential role of this gene during certain stages of mammary development. CDKN1A expression is indirectly induced by progesterone [57] and corticosterone [58], both of which were found increased, albeit at a later time point than CDKN1A, in our IUGR rats. CDKN1A promotes differentiation and inhibits proliferation via binding of PCNA [59], which was reduced in TEBs of our IUGR rats. Controversely, Fernandez-Twinn et al. [17] found an increase of PCNA protein expression, accompanied by an increase in CDKN1A mRNA expression in their IUGR model. These differences could be explained by the presence of additional factors which might influence PCNA regulation, especially by their continuous postnatal protein restriction until day 21 in their animal model. Interestingly, CDKN1A expression is known to be induced by Wilms tumor 1 (WT1) independently from p53 [60]. While Fernandez-Twinn et al. [17] did not detect changes in WT1 protein expression at 5 weeks of age in their model, we found a significant induction of WT1 expression (mRNA/protein) in the mammary glands of our IUGR animals. As a transcription factor, WT1 regulates key differentiation genes [61]. The cell type, its current state of development [61] and the ratio of WT1 isoform expression [62] may be crucial for WT1 to either promote or suppress tumor development. On the other hand, mammary tumors with high levels of WT1 have a poor prognosis and high levels of WT1 expression are frequently observed in cases of breast cancer that are estrogen and progesterone receptor negative [63]. WT1 expression is regulated by progesterone in a tissue dependent manner. So far the exact role of WT1 in the female reproductive system is not fully understood. In healthy endometrium progesterone induces WT1 isoforms which in turn leads to the differentiation into decidua [64], while in breast cancer cells progesterone analogs reduce WT1 expression thereby inducing differentiation [65]. As we examined mammary tissue without overt pathologic alterations, the observed induction of WT1 might point to premature differentiation, which is supported by the findings of our whole mount analyses showing smaller mammary glands and by the fact that an early increase in progesterone is detected in IUGR animals at day 28. In contrast to the results of our study, the mammary glands of IUGR rats investigated by Fernandez-Twinn et al. showed a proliferative phenotype with catch-up growth and no change in serum progesterone [12] nor mammary gland WT1 expression [17]. As this model differs from ours by its continuous postpartal malnutrition until day 21, such regimen might exert its influence on mammary gland development rather via changes in the actions of insulin and IGF-1 [45], than via alteration of ovarian steroid levels. In our IUGR model we showed WT1 induction in kidneys of male offspring [38], an organ which is dependent on epithelial-mesenchymal interactions in its postnatal development, similar to the mammary gland.

Another important pathway for mammary gland development (reviewed by [66]) and breast cancer development [67] is the ERK-1/-2 pathway. We found a significant reduction of mammary gland ERK-1/-2 activation in our IUGR rats at the beginning of ductal morphogenesis. ERK-1/-2 regulates context-dependent processes of cellular mammary gland growth and differentiation via downstream substrates, including regulators of transcription, apoptosis and steroid hormone receptors. Our finding could indicate a reduced local action of endocrine factors in mammary tissue of IUGR rats at day 21 and underlines the role of intrauterine malnutrition for this pathway. The fact, that the reduction in ERK-1/-2 activity was limited to day 21 only, might point to a change in endocrine milieu at day 28, which might be reflected by a significant increase in serum progesterone in our IUGR rats at this time-point. Growing evidence points to a cross-talk of progesterone and ERK-1/-2 action [68], [69]. Interestingly, mammary glands of IUGR animals fed a high-fat diet after puberty, show an increased phosphorylation of ERK-1/-2 and an induction of progesterone receptor at 4 months of age, which was associated with an increase in mammary tumor risk [17]. Similar findings were described for rats with high birth weight [70].

The finding of our in silico analysis that the downregulation of TGF-β1 becomes a central motive for cellular development at day 28 in the mammary gland seems an interesting future perspective, as TGF-β1, secreted by ductal epithelium, is the major local inhibitor of ductal elongation and lateral branching during mammary development (reviewed by [71]). In breast cancer, TGF-β1 can function as a tumor suppressor or promotor, dependent on the tumor stage (reviewed by [72]). Importantly, TGF-β1 not only acts on the TEB, but also determines the microenvironmental composition (stromal, immune and vascular cells) of the breast. There is evidence that these changes in tissue composition may supersede any tumor suppressive effects of TGF-β1 in epithelia [72]. We did not observe structural differences in our epithelia up to day 28. However, tertiary branching (induced by progesterone) takes place until day 35. Hypothetically, low local TGF-β1 in the LP group might facilitate the action of increased progesterone levels on lateral branching following day 28, leading to increased growth of the gland (as observed by [12]). Moreover, an altered mammary microenvironment might lead to a mal-response to DNA damage facilitating future cancerogenesis [72], as described by [12]. Noteworthy, a recent study found TGF-β1 signaling to be attenuated in the lungs of IUGR offspring, which may contribute to IUGR-associated lung disease [73].

Our study is limited in that we used mRNA from the entire mammary gland (mixed-cells-approach), which might have masked cell specific changes due to a potentially higher signal-to-noise ratio. While WT1 and CDKNA1 were of epithelial origin, gene array analysis could not distinguish between effects coming from the different mammary compartments. Some studies have carried out laser capture microdissection when cell-type specific gene expression profiles are necessary for an in depth evaluation [74]. However, our intent was to examine the overall response of the mammary gland to low protein diet.

Further limitations are related to the use of the IPKB database. As a manually updated resource based on current scientific literature, the attribution of functions to gene sets is constantly subject to change as the literature evolves. It is noteworthy, that IPKB-based relations found in our study are not exclusively mammary-specific, as IPA analysis returns relevant information obtained from other organ systems and in vitro studies.Taken together, we were able to show that IUGR influences the mammary transcriptome during ductal morphogenesis. We identified several differentially regulated growth factors (i.e. CDKN1A, WT1) and altered pathways involved in growth and differentiation (i.e. ERK-1/-2 and TGF-β1). The functional role of these changes for the mammary gland remains to be determined in future studies.

## Supporting Information

Figure S1 Determination of proliferation rate in terminal and buds (TEB) via PCNA stain: (A) Representative TEB with ◊  =  adipocytes, □  =  body cells, ○  =  cap cells, Δ  =  epithelial gland cells and (B) identification of PCNA positive cells (marked by yellow dots) per TEB.
(TIF)

Figure S2 PDF-files showing the summarized results of the online functional pathway analysis conducted with IPA ingenuity software. For a detailed view of the software output see Table S1.
(PDF)

Figure S3 PDF-files showing the summarized results of the online functional pathway analysis conducted with IPA ingenuity software. For a detailed view of the software output see Table S1.
(PDF)

Figure S4 Central role of transforming growth factor beta 1 (TGF-β1): (A) and (B) indicate differentially regulated genes of the TGF-β1 pathway at day 21 and day 28, respectively. Data was generated in silico using IPA Ingenuity software analysis. Multiple differentially regulated genes can be found in the TGF-β1 pathway in the mammary gland at day 28. Red =  up-regulated gene; green =  down-regulated gene; white =  fold-change value of 0.
(TIF)

Figure S5 Central role of ERK-1/-2 (marked by orange circle): Figure shows differentially regulated genes of the signaling network “carbohydrate metabolism, drug metabolism, small molecule biochemistry” at day 21. Data was generated in silico using IPA Ingenuity software analysis. Red =  up-regulated gene; green =  down-regulated gene; white =  fold-change value of 0.
(TIF)

Method S1 PDF-file containing a description of LC-Tandem-MS method, including Figure I A and B and 3 references.
(PDF)

Table S1 Spread-sheet listing detailed information on functional pathway analysis results retrieved from IPA Ingenuity in silico analysis for day 21 (sheet 1) and day 28 (sheet 2), as outlined in Figures S2 & S3.
(XLS)
