# Supplementary figures and images for: Detection of Expressional Changes Induced by Intrauterine Growth Restriction in the Developing Rat Mammary Gland via Exploratory Pathways Analysis
Source: PLoS One. 2014 Jun 23;9(6):e100504. doi: 10.1371/journal.pone.0100504 (PMC4067350; doi:10.1371/journal.pone.0100504)

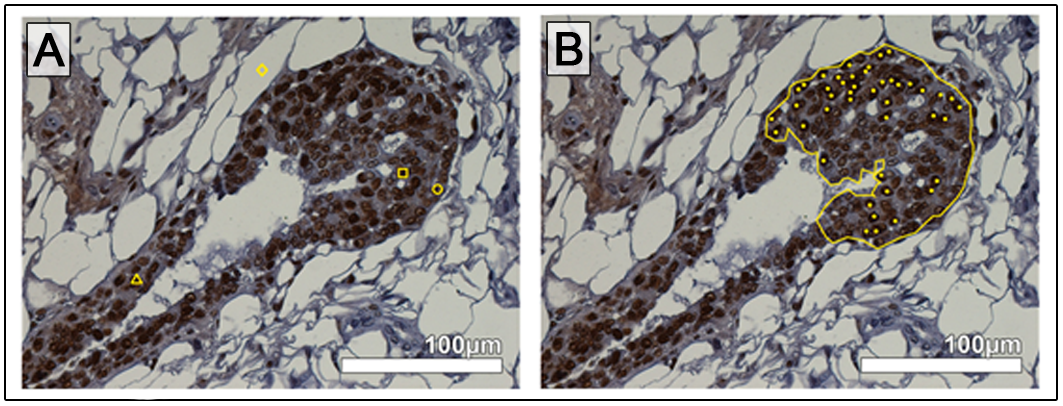

Supplement: Figure S1 — Determination of proliferation rate in terminal and buds (TEB) via PCNA stain: (A) Representative TEB with ◊ = adipocytes, □ = body cells, ○ = cap cells, Δ = epithelial gland cells and (B) identification of PCNA positive cells (marked by yellow dots) per TEB. (TIF) [file pone.0100504.s001.tif]

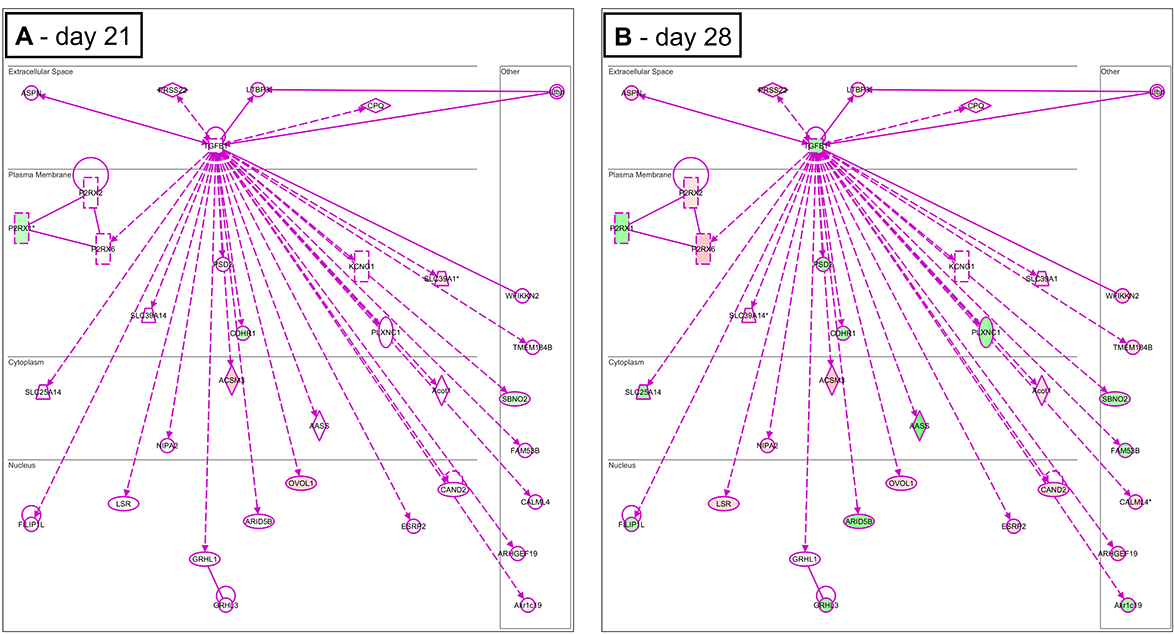

Supplement: Figure S4 — Central role of transforming growth factor beta 1 (TGF-β1): (A) and (B) indicate differentially regulated genes of the TGF-β1 pathway at day 21 and day 28, respectively. Data was generated in silico using IPA Ingenuity software analysis. Multiple differentially regulated genes can be found in the TGF-β1 pathway in the mammary gland at day 28. Red = up-regulated gene; green = down-regulated gene; white = fold-change value of 0. (TIF) [file pone.0100504.s004.tif]

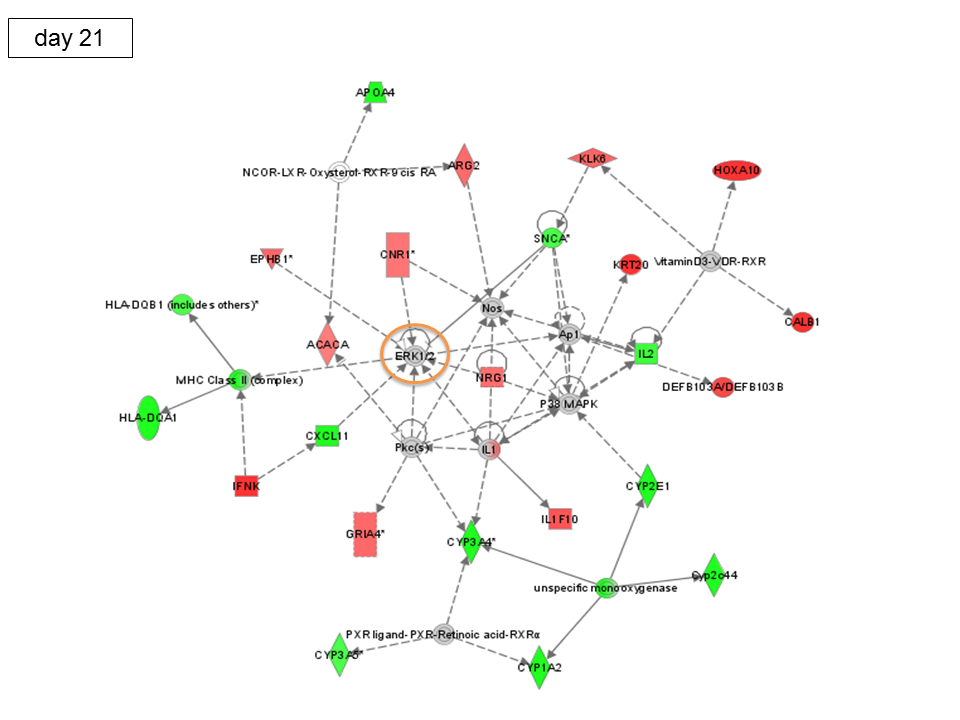

Supplement: Figure S5 — Central role of ERK-1/-2 (marked by orange circle): Figure shows differentially regulated genes of the signaling network “carbohydrate metabolism, drug metabolism, small molecule biochemistry” at day 21. Data was generated in silico using IPA Ingenuity software analysis. Red = up-regulated gene; green = down-regulated gene; white = fold-change value of 0. (TIF) [file pone.0100504.s005.tif]
