# Supplementary material for: Detection of Expressional Changes Induced by Intrauterine Growth Restriction in the Developing Rat Mammary Gland via Exploratory Pathways Analysis
Source: PLoS One. 2014 Jun 23;9(6):e100504. doi: 10.1371/journal.pone.0100504 (PMC4067350; doi:10.1371/journal.pone.0100504)
Supplement: Figure S2 — PDF-files showing the summarized results of the online functional pathway analysis conducted with IPA ingenuity software. For a detailed view of the software output see Table S1. (PDF) [file pone.0100504.s002.pdf]

Analysis Name: filtered d21 2 0.05 rat exp alltissues neu_2 alldatasource
Analysis Creation Date: 2013-06-24
Build version: 220217
Content version: 16542223 (Release Date: 2013-05-13)

Analysis settings

View

Reference set: SurePrint G3 Rat GE 8x60K Microarray

Relationship to include: Direct and Indirect

Includes Endogenous Chemicals

Optional Analyses:

Filter Summary:

Consider only molecules and/or relationships where

(species = Rat) AND

(confidence = Experimentally Observed)

Cutoff:

Fold Change = 2,000

p-value = 5,00E-02

Top Networks

ID	Associated Network Functions	Score

(c) 2000-2013 Ingenuity Systems, Inc. All rights reserved.

1	Lipid Metabolism, Molecular Transport, Small Molecule Biochemistry	16
2	Cell-To-Cell Signaling and Interaction, Nervous System Development and Function, Behavior	15
3	Cancer, Cellular Assembly and Organization, Cellular Compromise	2
4	Cell Death and Survival, Cellular Function and Maintenance, Small Molecule Biochemistry	2
5	Nucleic Acid Metabolism, Small Molecule Biochemistry, Cancer	2

(c) 2000-2013 Ingenuity Systems, Inc. All rights reserved.

Top Bio Functions

Diseases and Disorders

Name	p-value	# Molecules
Inflammatory Response	7,39E-05 - 4,64E-02	9
Cancer	1,34E-03 - 4,24E-02	9
Neurological Disease	4,90E-03 - 3,12E-02	6
Renal and Urological Disease	4,90E-03 - 2,66E-02	7
Skeletal and Muscular Disorders	1,02E-02 - 3,12E-02	2

Molecular and Cellular Functions

Name	p-value	# Molecules
Cell-To-Cell Signaling and Interaction	3,41E-04 - 4,64E-02	16
Cell Death and Survival	1,44E-03 - 4,64E-02	6
Drug Metabolism	1,44E-03 - 4,64E-02	5
Lipid Metabolism	1,44E-03 - 4,64E-02	12
Molecular Transport	1,44E-03 - 4,64E-02	17

Physiological System Development and Function

Name	p-value	# Molecules
Hematological System Development and Function	3,41E-04 - 4,64E-02	11
Immune Cell Trafficking	3,54E-03 - 4,64E-02	5
Nervous System Development and Function	4,13E-03 - 4,64E-02	11
Auditory and Vestibular System Development and Function	1,57E-02 - 1,57E-02	1
Behavior	1,57E-02 - 4,64E-02	2

(c) 2000-2013 Ingenuity Systems, Inc. All rights reserved.

Top Canonical Pathways

Name	p-value	Ratio
Nicotine Degradation III	4,59E-05	6/48 (0,125)
Melatonin Degradation I	4,59E-05	6/48 (0,125)
Superpathway of Melatonin Degradation	6,86E-05	6/52 (0,115)
Autoimmune Thyroid Disease Signaling	9,46E-05	5/34 (0,147)
Nicotine Degradation II	1,4E-04	6/57 (0,105)

Top Molecules

Fold Change up-regulated

Molecules	Exp. Value	Exp. Chart
NPHS2	↑15,206	
A1CF	↑13,886	
KRT35	↑8,849	
POU3F3	↑8,174	
GALR1	↑7,185	
UGT2B7	↑6,714	
WT1	↑5,316	
Idi2	↑5,069	
IFNK	↑4,682	
CALB1	↑3,917	

Fold Change down-regulated

Molecules	Exp. Value	Exp. Chart
TNFRSF17	↓-6,340	
CYP3A4	↓-6,030	
SULT2A1	↓-5,586	

(c) 2000-2013 Ingenuity Systems, Inc. All rights reserved.

INGENUITY® SYSTEMS

APOH	↓-5,337
RNASE3	↓-5,217
Mug1/Mug2	↓-5,110
HRG	↓-4,556
Klra5 (includes others)	↓-4,461
Try4/Try5	↓-4,420
Ugt2b	↓-4,097

Top Upstream Regulators

Upstream Regulator	p-value of overlap	Predicted Activation State
LEPR	6,70E-03	
GPRASP1	1,50E-02	
SREBF1	2,34E-02	
NR1I2	2,97E-02	

Top Tox Lists

Name	p-value	Ratio
Cytochrome P450 Panel - Substrate is a Xenobiotic (Human)	9,31E-05	4/16 (0,25)
Fatty Acid Metabolism	2,8E-04	8/108 (0,074)
Cytochrome P450 Panel - Substrate is a Xenobiotic (Rat)	6,76E-04	4/26 (0,154)
VDR/RXR Activation	1,07E-03	6/75 (0,08)
LXR/RXR Activation	1,19E-03	7/106 (0,066)

(c) 2000-2013 Ingenuity Systems, Inc. All rights reserved.

Top Tox Functions

Hepatotoxicity

Name	p-value	# Molecules
Glutathione Depletion In Liver	3,54E-03 - 1,60E-01	3
Liver Fibrosis	3,54E-03 - 3,54E-03	2
Hepatocellular Carcinoma	1,57E-02 - 1,57E-02	1
Liver Hyperplasia/Hyperproliferation	1,57E-02 - 1,57E-02	1
Liver Inflammation/Hepatitis	1,57E-02 - 1,57E-02	1

Nephrotoxicity

Name	p-value	# Molecules
Kidney Failure	4,90E-03 - 4,90E-03	2
Renal Transformation	3,12E-02 - 3,12E-02	1
Glomerular Injury	1,05E-01 - 1,05E-01	1
Renal Fibrosis	1,05E-01 - 1,05E-01	1
Renal Damage	1,60E-01 - 1,76E-01	4

(c) 2000-2013 Ingenuity Systems, Inc. All rights reserved.
