# Supplementary material for: Detection of Expressional Changes Induced by Intrauterine Growth Restriction in the Developing Rat Mammary Gland via Exploratory Pathways Analysis
Source: PLoS One. 2014 Jun 23;9(6):e100504. doi: 10.1371/journal.pone.0100504 (PMC4067350; doi:10.1371/journal.pone.0100504)
Supplement: Figure S3 — PDF-files showing the summarized results of the online functional pathway analysis conducted with IPA ingenuity software. For a detailed view of the software output see Table S1. (PDF) [file pone.0100504.s003.pdf]

IPA® Interactive pathway analysis of complex 'omics data

INGENUITY® SYSTEMS

Analysis Name: filtered d28 2 0.05 rat exp alltissues neu_2 alldatasource
Analysis Creation Date: 2013-06-24
Build version: 220217
Content version: 16542223 (Release Date: 2013-05-13)

Analysis settings

View

Reference set: SurePrint G3 Rat GE 8x60K Microarray

Relationship to include: Direct and Indirect

Includes Endogenous Chemicals

Optional Analyses:

Filter Summary:

Consider only molecules and/or relationships where

(species = Rat) AND

(confidence = Experimentally Observed)

Cutoff:

Fold Change = 2,000

p-value = 5,00E-02

Top Networks

ID	Associated Network Functions	Score
1	Drug Metabolism, Lipid Metabolism, Small Molecule Biochemistry	16

(c) 2000-2013 Ingenuity Systems, Inc. All rights reserved.

2	Cardiovascular System Development and Function, Hematological System Development and Function, Behavior	12
3	Organismal Injury and Abnormalities, Cardiovascular Disease, Psychological Disorders	3
4	Cell Morphology, Drug Metabolism, Hair and Skin Development and Function	2
5	Developmental Disorder, Embryonic Development, Hereditary Disorder	2

(c) 2000-2013 Ingenuity Systems, Inc. All rights reserved.

Top Bio Functions

Diseases and Disorders

Name	p-value	# Molecules
Connective Tissue Disorders	8,24E-03 - 1,91E-02	3
Inflammatory Disease	8,24E-03 - 4,97E-02	4
Skeletal and Muscular Disorders	8,24E-03 - 1,91E-02	3
Cancer	1,27E-02 - 1,31E-02	5
Cardiovascular Disease	1,27E-02 - 3,75E-02	1

Molecular and Cellular Functions

Name	p-value	# Molecules
Amino Acid Metabolism	8,12E-04 - 3,75E-02	4
Molecular Transport	8,12E-04 - 4,97E-02	7
Small Molecule Biochemistry	8,12E-04 - 4,97E-02	11
Cellular Development	1,55E-03 - 4,97E-02	6
Carbohydrate Metabolism	1,27E-02 - 4,97E-02	2

Physiological System Development and Function

Name	p-value	# Molecules
Skeletal and Muscular System Development and Function	4,25E-03 - 4,97E-02	5
Embryonic Development	1,05E-02 - 4,97E-02	5
Organ Development	1,05E-02 - 4,97E-02	5
Organismal Development	1,05E-02 - 4,97E-02	5
Tissue Development	1,05E-02 - 4,97E-02	8

(c) 2000-2013 Ingenuity Systems, Inc. All rights reserved.

INGENUITY® SYSTEMS

Top Canonical Pathways

Name	p-value	Ratio
Thyroid Hormone Metabolism II (via Conjugation and/or Degradation)	2,24E-03	3/27 (0,111)
Phototransduction Pathway	2,9E-03	4/47 (0,085)
Thiamin Salvage III	1,27E-02	1/1 (1)
cAMP-mediated signaling	1,48E-02	7/204 (0,034)
Melatonin Degradation I	1,6E-02	3/48 (0,062)

Top Molecules

Fold Change up-regulated

Molecules	Exp. Value	Exp. Chart
MYF6	↑12,118	
MYOD1	↑9,330	
IGSF5	↑8,386	
TPK1	↑8,079	
CADPS	↑7,785	
PITX2	↑6,851	
PPP1R3A	↑6,619	
GZMB	↑5,963	
KCNJ10	↑5,749	
CHRM5	↑5,735	

Fold Change down-regulated

Molecules	Exp. Value	Exp. Chart
BMP10	↓-8,574	
GLB1L3	↓-3,993	
CNGA1	↓-3,362	

(c) 2000-2013 Ingenuity Systems, Inc. All rights reserved.

NR1D1	↓-3,221
SVOP	↓-3,126
MYH6	↓-2,515
OLFM3	↓-2,513
TNR	↓-2,489
CCR4	↓-2,464
DNAJC27	↓-2,382

Top Upstream Regulators

Upstream Regulator	p-value of overlap	Predicted Activation State
TNFRSF1A	5,11E-04	
Pkc(s)	5,76E-03	
THRA	1,31E-02	
ARG1	1,31E-02	
TNFRSF1B	2,61E-02	

Top Tox Lists

Name	p-value	Ratio
Cytochrome P450 Panel - Substrate is a Xenobiotic (Mouse)	3,12E-02	2/23 (0,087)
Nongenotoxic Hepatocarcinogenicity Biomarker Panel	3,12E-02	2/22 (0,091)
CAR/RXR Activation	3,95E-02	2/26 (0,077)
Cytochrome P450 Panel - Substrate is a Xenobiotic (Rat)	4,25E-02	2/26 (0,077)
Reversible Glomerulonephritis Biomarker Panel (Rat)	4,55E-02	2/27 (0,074)

(c) 2000-2013 Ingenuity Systems, Inc. All rights reserved.

INGENUITY® SYSTEMS

Top Tox Functions

Cardiotoxicity

Name	p-value	# Molecules
Cardiac Hypertrophy	1,27E-02 - 7,36E-02	2
Cardiac Arrythmia	3,75E-02 - 3,75E-02	1
Cardiac Damage	3,75E-02 - 3,75E-02	1
Tachycardia	3,75E-02 - 3,75E-02	1

Hepatotoxicity

Name	p-value	# Molecules
Liver Proliferation	1,27E-02 - 1,27E-02	1
Liver Necrosis/Cell Death	2,73E-01 - 2,73E-01	1

Nephrotoxicity

Name	p-value	# Molecules
Renal Necrosis/Cell Death	7,36E-02 - 7,36E-02	1

(c) 2000-2013 Ingenuity Systems, Inc. All rights reserved.
