# Supplementary material for: Detection of Expressional Changes Induced by Intrauterine Growth Restriction in the Developing Rat Mammary Gland via Exploratory Pathways Analysis
Source: PLoS One. 2014 Jun 23;9(6):e100504. doi: 10.1371/journal.pone.0100504 (PMC4067350; doi:10.1371/journal.pone.0100504)
Supplement: Method S1 — PDF-file containing a description of LC-Tandem-MS method, including Figure I A and B and 3 references. (PDF) [file pone.0100504.s006.pdf]

Supplementary Methods 1

Liquid Chromatography Tandem Mass Spectrometry (LC-Tandem MS)

Sample preparation

Steroid profiling was performed by LC-Tandem MS, as previously described by us in detail [1, 2]. Materials came from Merck (Darmstadt, Germany), unless stated otherwise. Blood was drawn via heart puncture at the time of sacrifice. Blood was stored at 4°C overnight, centrifuged at 3500 U/min for 15 min and serum was stored at -80°C until further analysis. Our prequels showed that serum levels of pregnenolone, 17OH-pregnenolone, 17OH-progesterone, 21OH-progesterone, androstenedione, testosterone and estradiol were below the range of detection, while serum levels of dehydro-epiandrosterone (DHEA), dehydro-epiandrosterone sulfate (DHEAS) lay close to the detection limit. Hence, the levels of the hormones progesterone, corticosterone, deoxycorticosterone, dehydrocorticosterone, DHEA and DHEAS were determined in 100µl serum. Cortisol-d4, DHEAS-d5, corticosterone-d8 and progesterone-d9 (in 2 mM ammonium acetate and methanol 1:1 at a concentration of 150µg/l) served as internal standards. Maternal serum served as a positive control. 7 calibrator standards were used per run. The substrates were stored at 4°C until usage. Incubation with standards (20µl) was performed at room temperature for 30 min, followed by precipitation with 200µl of a 1:1 H2O-solution of methanol and ZnSO4 (50 g/l). After incubation of 15 min at 4°C samples were spun down for 10 min at 18°C at 15.000 U/min. Supernatants at a final volume of 250µl were transferred onto a microtiter plate (Greiner BIO-ONE, Frickenhausen, Germany) for further massspectrometrical analysis at 15°C.

Chromatographic conditions

LC-Tandem MS was performed using an online SPE-HPLC-MS/MS assay developed by Rauh et al. [1, 2] allowing quantitative analysis of steroid hormones in 100 µL serum with atmospheric pressure chemical ionization in the positive ion mode. For online SPE was a Chromolith extraction column (4.6 x 50 mm) coupled to a Chromolith HPLC column (RP-18e, 100 x 3 mm, Merck) was used. The autosampler (HTC PAL, CTC Analytics) was fitted with a 250 µL sample loop. HPLC MS/MS was performed with a Shimadzu LC-20AD HPLC unit and a quaternary pump (HPLC1200 series, Agilent Technologies, Waldbronn, Germany). Samples were washed methanol (5%) and eluted with 2 mM ammonium acetate/MeOH (50:50, v/v) onto the analytical column in back-flush. Mobile phase A consisted of MeOH and water (with 2.0 mM ammonium acetate) in a mixture of 90:10 (v:v). The mixture of mobile phase B consisted of water (with 2.0 mM ammonium acetate; pH set to 4.5 via acetic acid)

and MeOH in a ratio of 95:5 (v:v). The total flow rate of the binary gradient module was maintained at 1 ml/min. The starting conditions (50% A) were raised via a linear gradient to 56% A after 1.0 min in 3.8 min, followed by linear gradient to 90% A in 1.7 min and a return to initial conditions after 7.5 min. The SPE-HPLC-MS/MS analysis time per sample was 9 min total. Column temperature was set at 40 °C. The injection volume was 200 µL. MRM based mass spectrometric detection was performed with a 4000QTrap® triple quadrupole mass spectrometer (AB Sciex, Toronto, Canada) equipped with an APCI source in positive mode. For sample analysis multiple-reaction monitoring mode was used. For Quantification transitions outlined in Figure I were chosen. Mass spectrometric parameters were as follows: Collision assisted dissociation Gas (CAD) at 7, Curtain Gas (CUR) at 30, sheath Gas (GS1) at 55, nebulizer current (NC) at 5.0, temperature (TEM) at 550 °C and resolution at unit. Figure I lists the intensity, multiple reaction monitoring results and retention time parameters for the respective hormones. Samples quantification was performed via linear regression and 1/x-weighing, based on the 7 calibrator samples as previously described [3].

A

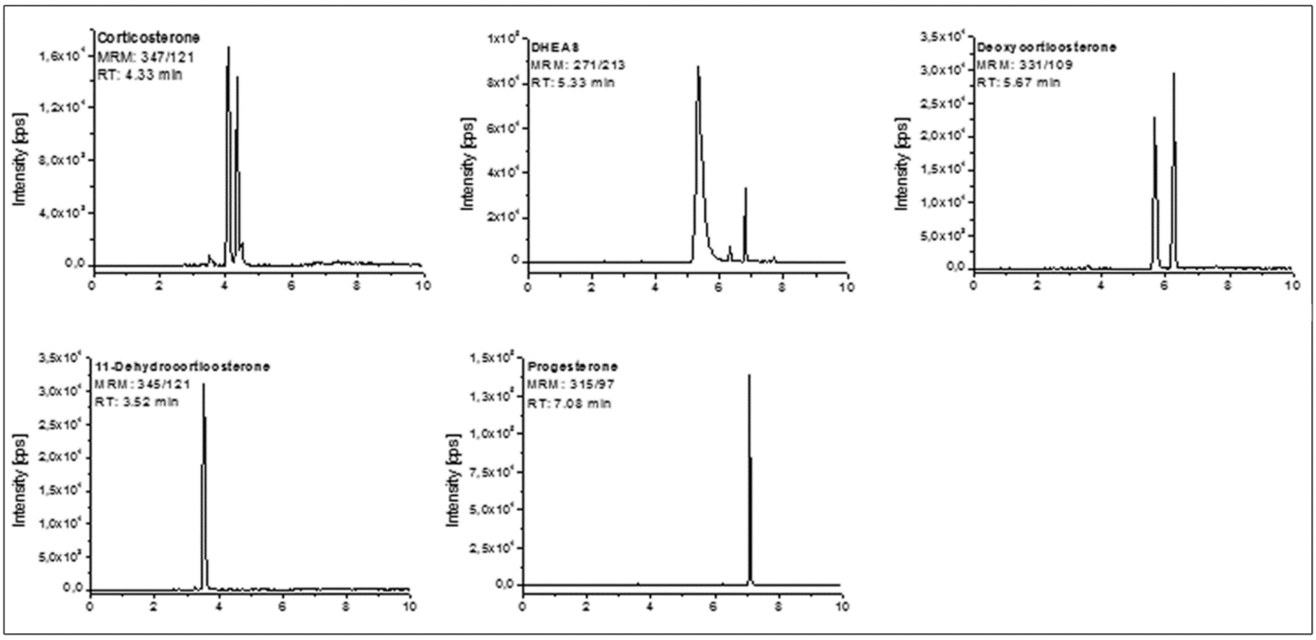

B

Steroid	MW	Ion	CAS-Nummer	MRM	MRM-Q	IS
Corticosterone	346.2	[M+H]+	50-22-6	347/121	347/97	355/125
Dehydrocorticosterone	344.2	[M-H]+		345/121	345/	355/125
Deoxycorticosterone	330.2	[M+H]+	64-85-7	331/109	331/97	339/113
Progesterone	314.2	[M+H]+	57-83-0	315/97	315/109	339/113
DHEA	288.2	[M-H2O+H]+	53-43-0	271/213	271/197	339/113
DHEAS	368.2	[M-SO4-H2O+H]+	651-48-9	271/213	271/197	273/213

Figure I) LC-Tandem MS: A) Representative display of mass spectrometric intensity in relation to time (min). B) Properties of steroidal hormones. Legend: cps = counts per second (intensity); MRM = multiple reaction monitoring; RT = retention time; MW= molecular weight (in Da).

References

1 Rauh M (2009) Steroid measurement with lc-ms/ms in pediatric endocrinology. Mol Cell Endocrinol 301:272-281.

2 Rauh M, Groschl M, Rascher W, Dorr HG (2006) Automated, fast and sensitive quantification of 17 alpha-hydroxy-progesterone, androstenedione and testosterone by tandem mass spectrometry with on-line extraction. Steroids 71:450-458.

3 Fahlbusch FB, Ruebner M, Rascher W, Rauh M (2013) Combined quantification of corticotropin-releasing hormone, cortisol-to-cortisone ratio and progesterone by liquid chromatography-tandem mass spectrometry in placental tissue. Steroids 78:888-895.
